# Impact of Heterotopic Ossification on Functional Recovery in Acute Spinal Cord Injury

**DOI:** 10.3389/fncel.2022.842090

**Published:** 2022-02-09

**Authors:** Steffen Franz, Lukas Rust, Laura Heutehaus, Rüdiger Rupp, Christian Schuld, Norbert Weidner

**Affiliations:** Spinal Cord Injury Center, Heidelberg University Hospital, Heidelberg, Germany

**Keywords:** spinal cord injury, functional recovery, heterotopic ossification, complication, ADL, activities of daily living, independence, SCI

## Abstract

**Objective**: In spinal cord injury (SCI), heterotopic ossification is a frequent secondary complication, commonly associated with limited range of motion of affected joints, which could lead to secondary disability in activities of daily living. Additionally, heterotopic ossifications might challenge the effect of regeneration-promoting therapies on neurological and functional recovery. This study evaluated the impact of heterotopic ossification on clinical recovery within the first year after SCI.

**Methods**: The study was conducted as a monocentric longitudinal paired cohort study. Recruitment was based on consecutive sampling in the framework of the European Multicenter about Spinal Cord Injury (EMSCI). Recovery profiles were determined using standardized neurological and functional clinical assessments within the 1st year following SCI. All study participants underwent at least two comprehensive standardized neurological and functional clinical examinations according to the International Standards for Neurological Classification of SCI and the Spinal Cord Independence Measure, respectively. Data regarding the diagnosis and treatment of heterotopic ossification were obtained by reviewing the patient medical records. The most similar “digital twin” from the entire EMSCI database were matched in terms of age, acute neurological and functional status to each individual with SCI, and heterotopic ossification.

**Results**: Out of 25 participants diagnosed with heterotopic ossification, 13 individuals were enrolled and matched to control individuals. Most individuals presented with motor complete injury (75%). Ossifications were most frequently located at the hip joints (92%) and mainly occurred within the first 3 months after SCI. Individuals with heterotopic ossification achieved around 40% less functional improvement over time compared to their matched counterparts, whereas neurological recovery was not altered in individuals with SCI and heterotopic ossification.

**Conclusion**: Heterotopic ossification—a common complication of SCI—unfavorably affects functional recovery, which in the end is most relevant for the best possible degree of independence in activities of daily living. Upon presentation with heterotopic ossification, neurological improvement achieved through potential restorative therapies might not translate into clinically meaningful functional improvement. Diagnostic algorithms and effective early prevention/treatment options for heterotopic ossification need to be established to ensure the best possible functional outcome.

**Clinical Trial Registration**: NCT01571531 (https://clinicaltrials.gov).

## Introduction

The extent of neurological impairment after spinal cord injury (SCI) represents the most powerful predictor of spontaneous sensorimotor and autonomic improvements and subsequently functional recovery. Patients with initially motor complete SCI (AIS-A or AIS-B) display very limited sensorimotor improvements with the inability to restore for example standing and walking function, whereas patients with initially motor incomplete SCI (AIS-C or AIS-D) typically show substantial neurological and functional recovery (Kirshblum et al., 2021a). The aim of restorative therapeutic strategies such as stem cell transplantation or pro-regenerative drug administration is to expedite sensorimotor and autonomous improvement beyond natural recovery. While neurological recovery mainly depends on the integrity of its neural underpinning within the spinal cord, functional recovery referring to mobility, self-care, bowel, bladder, and respiratory function, requires timely and proper administration of rehabilitative interventions (Richard-Denis et al., 2018). A number of secondary complications arising after SCI, e.g., infections, spasticity, pain conditions, pressure injuries, and heterotopic ossification (HO), might negatively affect functional recovery, which is commonly assessed in a standardized fashion with the Spinal Cord Independence Measure (SCIM; Catz et al., 1997; Catz and Itzkovich, 2007; Itzkovich et al., 2007). For example, the presentation with advanced pressure injuries in patients with acute SCI has been shown to impair functional outcome (SCIM score; Donhauser et al., 2020).

Heterotopic ossification (HO) is a complication affecting soft tissues, which can occur after total arthroplasty, traumatic brain injury, or SCI (Déjérine and Ceillier, 1918; Garland, 1991a; Taly et al., 1999). In SCI, it mostly emerges during the first months after SCI below the level of injury (Garland, 1991a; van Kuijk et al., 2002). Most frequent localizations of HO in SCI are the proximal joints, particularly the hip joints (Garland, 1991a; Ranganathan et al., 2015). Around one-fifth of individuals with SCI have been described to present with HO (van Kuijk et al., 2002; Sakellariou et al., 2012; Ranganathan et al., 2015). HO occurs more frequently at younger ages, in men, after cervical or high thoracic traumatic SCI, and typically in more pronounced injury severities (Wittenberg et al., 1992). HO is discussed to favor secondary complications such as urinary tract infections, pneumonias, pressure injuries, and thromboembolic events (Wittenberg et al., 1992; Sakellariou et al., 2012). HO can severely affect the range of motion of affected joints and thus has a great potential to significantly affect the functional outcome, quality of life, and overall healthcare burden (Dryden et al., 2005; Cipriano et al., 2009; Craven et al., 2012). Potentially effective restorative and/or rehabilitative treatments could be challenged by HO to an extent that the gain of neurological recovery by the intervention will not translate into clinically meaningful function (Franz et al., 2012; Lu et al., 2012; Kucher et al., 2018).

While algorithms for early detection and diagnosis of HO are steadily improved (Brooker et al., 1973; Freed et al., 1982; Bressler et al., 1987; Pistarini et al., 1995; Wick et al., 2005; Rosteius et al., 2017) and different (prophylactic) treatment options are being evaluated (Banovac and Gonzalez, 1997; Meiners et al., 1997; Aubut et al., 2011; Genet et al., 2011; Museler et al., 2017), a detailed analysis of HO-associated consequences for the clinical outcome has yet to be determined (Garland, 1991a, b). Thus, this study is intended to clarify to which extent HO affects neurological and functional capabilities over the first year after injury.

## Material and Methods

### Study Design, Setting and Participants

This longitudinal paired cohort study was conducted in the framework of the “European Multicenter Study about Spinal Cord Injury” (EMSCI) at the Spinal Cord Injury Center Heidelberg, Germany (Curt et al., 2004). The study protocol was approved by the ethics committee of the Heidelberg University Hospital (S-188/2003) and registered at ClinicalTrials.gov (Register-no. NCT01571531). The present study is reported according to the guidelines entitled “Strengthening the Reporting of Observational studies in Epidemiology” (STROBE; Vandenbroucke et al., 2007; von Elm et al., 2007). Before enrolment, informed consent was obtained from all study participants. All participants of EMSCI enrolled from July 2002 to January 2020 were considered for data analyses.

EMSCI aims to include all eligible patients with acute traumatic or single event ischemic SCI according to consecutive sampling. Exclusion criteria comprise nontraumatic cause of SCI (except for single event ischemic incidences), impaired capabilities of cooperation or giving informed consent, peripheral nerve lesions above the level of the spinal lesion, medical history of polyneuropathy, and additional traumatic brain injury. Individuals with SCI who were assigned to the cohort of participants with HO were only assessed at the SCI Center at Heidelberg University Hospital. Participants assigned to the paired control group were identified within the whole EMSCI network. Within EMSCI, initial assessments must be performed within the first 6 weeks after injury. All study participants undergo recurrent comprehensive clinical examinations in defined time windows (up to day 40, between day 70 and 98, from day 150 to day 186, and from day 300 to day 546 after SCI) within the first year after injury. These comprise neurological examinations according to the “International Standards for Neurological Classification of Spinal Cord Injury” (ISNCSCI; Kirshblum et al., 2011; American Spinal Injury Association, 2019) and functional tests, such as the Spinal Cord Independence Measure (SCIM; Catz et al., 1997; Catz and Itzkovich, 2007). Data were collected by expertly trained examiners to ensure high quality standards (Curt et al., 2004; Schuld et al., 2013; Franz et al., 2022). Data collection and management were coordinated by means of the in-house established EMSCI database (Rupp et al., 2005).

During the study period, all EMSCI study participants from the SCI Center at Heidelberg University Hospital were included in this retrospective data analysis of HO. If at least ISNCSCI and SCIM assessments were completely conducted at the early stage (0–40 days after injury) as well as the late stage (150–546 days after injury) of SCI as previously published (Prang et al., 2021).

### Diagnosis of Heterotopic Ossification

**Figure 1:**
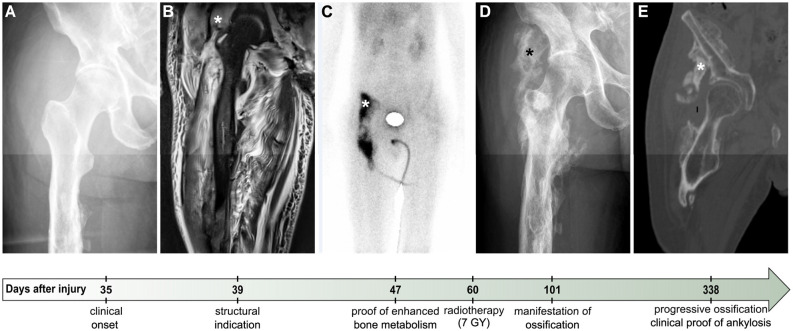
Exemplary clinical course of severe Heterotopic Ossification (ID 13). Plain X-ray **(A,D)**, MRI **(B)**, scintigraphy **(C)**, and CT **(E)**, in chronological sequence of the clinical course from the onset of symptoms to the decision on surgical resection of HO. “Days after injury” denote the time elapsed since SCI. Depicted stars (*) point to areas of ossification. Four days after first clinical indications of potential HO medical diagnostics were initiated (day 39 after SCI), with plain X-ray still lacking reliable proof of ossification **(A)**. On the same day, MRI showed suspicious but rather nonspecific diffuse T2 hyperintense muscle signal behavior in the vicinity of the femoral head **(B)**. Forty-seven days after SCI, enhanced bone metabolism detected by scintigraphy confirmed the previously suspected diagnosis of HO **(C)**. Despite a performed single-time radiotherapy with 7 Gy on day 60, a clinically relevant ossification was proven by CT more than 2 months after the onset of symptoms **(D)**. Ankylosis occurred roughly one year after SCI **(E)** and led to a surgical resection of the ossification (day 408 after SCI), immediately after a second single radiotherapy with 7 Gy the day before.

Heterotopic ossification as a secondary diagnosis (according to ICD-10) during the first year after SCI led to an assignment of individuals to the HO cohort. Besides relevant clinical symptoms such as redness, swelling, restricted range of motion (Citak et al., 2016), elevated serum levels of alkaline phosphatase (AP), and acute-phase-marker C-reactive protein (CRP; Singh et al., 2003; Estrores et al., 2004), diagnosis of HO was confirmed by plain X-ray, computed tomography, ultrasound, MRI, and 3-phase-bone technetium-99 m scintigraphy (Figure 1; Brooker et al., 1973; Freed et al., 1982; Bressler et al., 1987; Pistarini et al., 1995; Wick et al., 2005). The extent of HO in each participant was classified according to the Brooker stages based on available imaging results (Brooker et al., 1973).

### Clinical Examination and Quantification of Neurological Outcome

At each exam stage, the neurological assessment was done according to ISNCSCI (Rupp et al., 2021a). This examination includes a standardized motor examination of five upper and lower extremity key muscles and the assessment of two sensory modalities at 28 key sensory points on each side of the body. Based on this, the sensory, motor, and neurological levels of injury, as well as the severity of the SCI graded by the ASIA Impairment scale (AIS), are determined (American Spinal Injury Association, 2015).

Sum scores were calculated for each examination step and side of the body: “upper extremity motor score” (UEMS, maximum = 5 key muscles × 5 max. motor score × body sides = 50 points), “lower extremity motor score” (LEMS, maximum = 5 key muscles × 5 max. motor score × body sides = 50 points), “total light touch score” (TLT, maximum = 28 dermatomes × 2 max. sensory score × 2 sides of the body = 112 points), “total pin-prick score” (TPP, maximum = 28 dermatomes × 2 max. sensory score × 2 sides of the body = 56 × 2 = 112).

For more detailed information on the scoring, scaling and classification process according to ISNCSCI please see Rupp et al. (2021a).

### Quantification of Functional Outcome and Independence

The functional capabilities of participants were assessed based on the SCIM (Catz et al., 2007; Itzkovich et al., 2007). The SCIM is a measure of caregiver and assistive device independence in individuals with SCI. It covers the most important aspects of daily living. In EMSCI, an outdated version (SCIM II) was used up until 2007, followed by the current version (SCIM III) from 2007 (Catz et al., 1997; Catz and Itzkovich, 2007). SCIM II and III are compatible on the sub-total level. Sub-total scores are calculated for three different general domains of daily living: (1) “Self Care” (maximum score of 20), (2) “Respiration and Sphincter Management” (maximum score of 40), and (3) “Mobility” (maximum score of 40). Finally, the sum of all scores is defined as the total sum score (maximum score of 100, meaning complete independence).

### Matching Procedure

For evaluation of adequate matching partners, the whole EMSCI database was systematically queried applying an iterative approach with margins of matching criteria as narrow as possible being applied. The following matching criteria were applied only if corresponding EMSCI time frames were available in the early stage (either 0–14 or 15–40 days after injury) *and* in the late stage (either 150–186 or 300–546 days after injury): (1) complete ISNCSCI and SCIM assessment within 40 days after injury, (2) identical AIS, (3) neurological level of injury (NLI) ±1 spinal segment, (4) maximum difference of UEMS and LEMS of ±7 points each, (5) divergence of maximum ±4 points in SCIM total scores. In the case of multiple competing matching partners, the one being most akin to the HO-participant was selected. In this process, the lowest possible difference in initial SCIM scores was the determining factor between potential matching partners, followed by the best possible similarity in initial motor function (UEMS/LEMS). In case of persisting ambiguity, the next step was to ensure that the age difference was as small as possible without a fixed cut-off. As the final step in the decision-making process, the gender of the potential matching partners was considered. As the diagnosis of HO is not routinely documented in EMSCI, it is conceivable that a few individuals in the control group were accidentally affected by HO.

### Statistical Analysis

Processing (McKinney, 2010), statistics (Virtanen et al., 2020), and presentation (Hunter, 2007) of data were performed using Python Data Science Stack. Due to non-parametric distribution of data, one-sided Wilcoxon signed rank test for matched samples (zero method: “zplit”) was used to determine potential differences in neurological and functional recovery. A threshold of *p* < 0.05 was defined for statistical significance.

## Results

**Figure 2:**
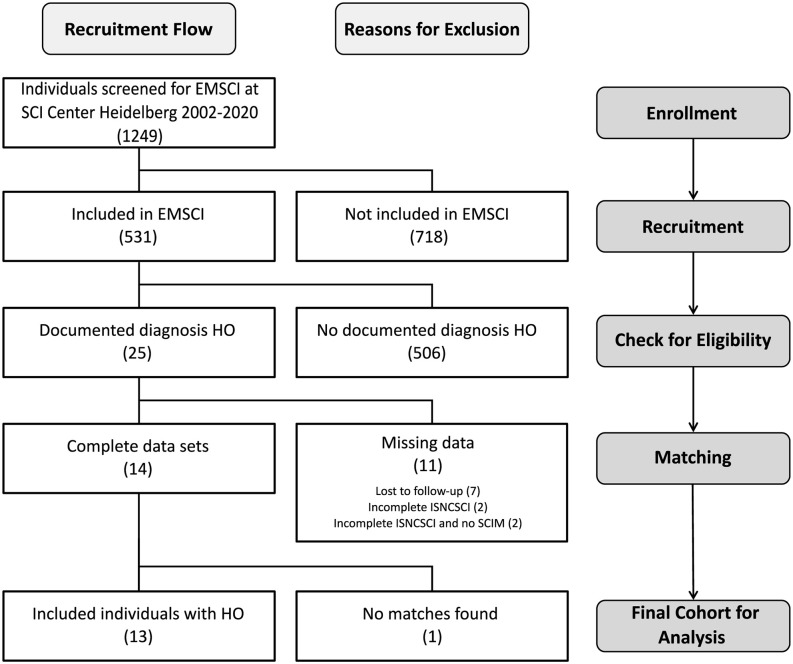
Flow diagram of recruitment process at each stage of the study. Abbreviations: HO, heterotopic ossification; EMSCI, European Multicenter Study about Spinal Cord Injury; ISNCSCI, International Standards for Neurological Classification of Spinal Cord Injury; SCIM, spinal cord independence measure.

Of 531 patients from the SCI center at Heidelberg University Hospital enrolled in EMSCI, 25 participants were diagnosed with HO in the 1st year after SCI. Of these, 11 had to be excluded (median age 36 years) from the analysis due to missing data in ISNCSCI, seven of which were characterized by a severely limited range of motion of HO-affected joints within the first 6 months after injury (6× at the hip joint limited to <90° flexion and 1× elbow joint limited to <80° flexion with a median time since the injury to HO diagnosis of 51.5 days, IQR 37.75–60.75). For example, this may have led to “not testable” key muscles and subsequently “not determinable” LEMS according to ISNCSCI (Figure 2). Fourteen individuals could be identified for whom a complete dataset was available, i.e., one early (up to 40 days after injury) and one late (150–546 days after injury) ISNCSCI and SCIM assessment. For 13 of these 14 individuals with HO (two females and 11 males, median age 56 years, five AIS A, four AIS B, three AIS C, one AIS D), the identical number of matched partners as controls was identified in the EMSCI database (three females and 10 males, median age 40 years; no significant difference between groups regarding age *p* = 0.12), leading to five mixed and eight male pairs. The matching accuracy for the 13 subjects with HO is reflected by similar baseline characteristics without significant differences regarding relevant ISNCSCI parameters (Tables s 2, 3). The median time from onset of SCI to the confirmed diagnosis of HO was 55 days (IQR 46.0–89.5). When HO was suspected, both AP and CRP showed elevated values with a median of 163 (IQR = 114–305) U/l (normal: 35–105 for females and 40–130 U/l for males) for AP and 22 (IQR = 17–39) mg/l (normal: <5 mg/l) for CRP. The diagnosis was confirmed by radiological workup (X-ray or computed tomography) in all but two cases. In these two cases again, MRI, supplemented by either ultrasound or scintigraphy, supported the HO diagnosis. HO was most frequently located at the femur and hip (92%, *n* = 3 + 9 = 12), respectively. One individual with cervical SCI developed HO at the shoulder. A detailed illustration of the diagnostic workflow is presented in 1.

**Table 1:** Individual characteristics of study participants related to spinal cord injury and initial management of heterotopic ossification.

ID	Gender	Age [years]	NLI	AIS	Cause	Localization	Brooker stage	TSI to HO [days]	Diagnostics							
									Abnormal AP/CRP	Ultrasound	MRI	Scinti	X-Ray	CT	TSD to 7 Gy Radiation [days]	TSD to Surgical Intervention [days]
**01**	m	33	T6	A	I	both hips	n.a.	56	yes/yes	no	no	no	yes	yes	no	1,414
**02**	m	74	T3	B	T	left femur	n.a.	24	n.a./n.a.	no	no	no	yes	yes	0	no
**03**	m	63	C5	A	T	left hip	n.a.	47	yes/yes	yes	yes	no	no	no	10	no
**04**	m	35	T3	B	I	left hip	n.a.	63	n.a./n.a.	yes	yes	no	yes	no	33	no
**05**	f	43	C5	C	T	left shoulder	n.a.	106	n.a./n.a.	no	no	no	yes	yes	27	no
**06**	m	65	C5	B	T	right femur	n.a.	120	no/no	no	no	no	yes	yes	14	no
**07**	m	48	C1	C	T	both hips	III	43	yes/yes	no	no	no	no	yes	16	202
**08**	m	57	C4	B	T	left hip	I	135	yes/yes	no	no	no	yes	no	21	no
**09**	m	66	C2	A	T	left hip	I	54	yes/yes	no	yes	no	yes	yes	29	no
**10**	m	68	C3	C	T	left hip	I	61	no/yes	no	no	no	yes	no	no	no
**11**	m	38	T4	A	T	both hips	IV	41	yes/yes	no	yes	yes	no	no	28	no
**12**	f	18	C4	D	T	right femur	II	84	no/yes	no	no	no	yes	no	no	no
**13**	m	56	T2	A	T	right hip	IV	47	yes/yes	no	yes	yes	yes	yes	13	361
Median								56							18.5	
(IQR)								(47–84)							(13.25–27.75)	

*Abbreviations: AIS, American Spinal Injury Association Impairment Scale; AP, alkaline phosphatase; CRP, C-reactive protein; CT, computed tomography; HO, heterotopic ossification; IQR, interquartile range; I, ischemia; MRI, magnetic resonance imaging; n.a., not available; NLI, Neurological Level of Injury; Scinti, scintigraphy; T, trauma; TSD, time since diagnosis (of HO); TSI, time since injury*.

**Table 2:** Detailed comparison of motor (sum) scores between individuals with heterotopic ossification and matched controls.

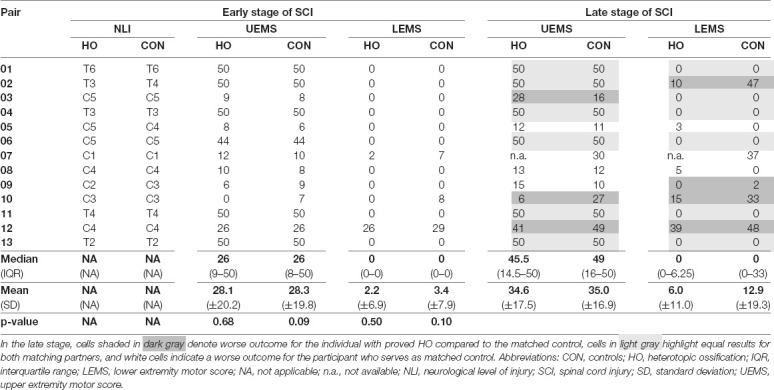

**Table 3:** Detailed comparison of total SCIM and its subscales between individuals with heterotopic ossification and matched controls.

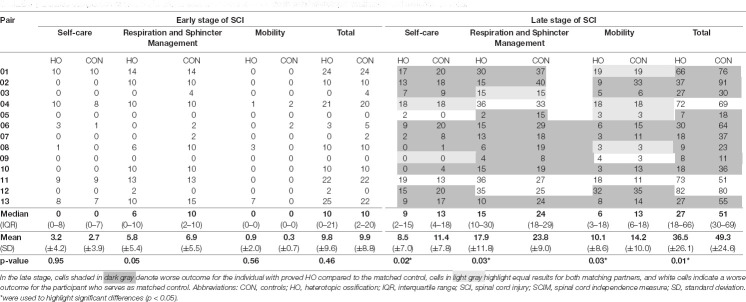

### Neurological Recovery Unaltered by Heterotopic Ossification

Individuals with and without HO—starting with similar if not identical degrees of motor impairment due to the narrow matching process—displayed comparable motor recovery in terms of UEMS and LEMS at the late stage (Table 2). Key muscles in the arms added up to a median UEMS of 45.5 (IQR = 14.5–50.0) in subjects with HO vs. 49.0 (16.0–50.0, *p* = 0.5; Table 2) in the control group. The same applies to the LEMS, where no differences between the groups are seen, even in the late stages after SCI (*p* = 0.1).

Total light touch scores did not differ in either the early and the late stage of SCI (*p*_early_ = 0.87, *p*_late_ = 0.58; Supplementary Table 1), whereas total pin-prick scores differed consistently in both groups with matched controls displaying a median of 1 and 3 points higher scores in the acute as well as chronic stage (*p*_early_ = 0.004, *p*_late_ = 0.02; Hales et al., 2015).

### Heterotopic Ossification Associated With Impaired Functional Recovery

As opposed to motor recovery, functional recovery as assessed by SCIM revealed differences in outcomes between the two investigated cohorts. While the total SCIM score including all subscales did not differ in the early stage, the median SCIM total score of individuals with HO was 27 (IQR 18–66) points, thus 47.1% lower (*p* = 0.01) in the late stage of SCI as compared to individuals of the control group who presented with a median total SCIM of 51 (IQR 30–69) in the late stage (Table 3). Notably, at the end of the observation period, all subscales of the SCIM showed relevant differences between both groups, albeit to a varying extent (Table 3). The self-care subscale median was 4 points lower in the HO group—corresponding to a 30.8% worse outcome (*p* = 0.02). The median of the respiration and sphincter management subscale was 9 points lower—corresponding to a 37.5% worse outcome (*p* = 0.03), while the median mobility subscale was 7 points lower—corresponding to 53.8% worse outcome (*p* = 0.03).

## Discussion

Neurological recovery—either naturally occurring or promoted by eventually established interventions and/or restorative therapies—represents the foundation for the restoration of physical abilities, e.g., ambulation or grasping function. However, neurological recovery does not transfer automatically in a meaningful functional gain and can be negatively affected by secondary complications of SCI. As the majority of secondary complications are reversible, e.g., pneumonia, thrombosis, or various pain conditions, they are expected to not interfere with achieving the highest possible functional outcome in the long run. In contrast, HO cannot easily be reversed and has the potential to permanently impair the translation of neurological recovery into functional improvement and independence in activities of daily living.

The present study demonstrates that despite a comparable motor recovery, the clinical manifestation of HO predisposes for unfavorable functional outcomes in individuals with SCI as determined by the SCIM assessment. The relatively large effect size supports the confidence in this observation despite a (relatively) small sample size. The limited number of individuals with SCI currently included in EMSCI did not allow to add both age and gender as matching criteria in the present study. The age in the HO cohort, although not significantly higher, may nevertheless have contributed to the observed differences in functional outcomes (Jakob et al., 2009; Kaminski et al., 2017; Geuther et al., 2019; Kirshblum et al., 2021b; Wichmann et al., 2021). Gender, which was not equally distributed in both groups, has not been identified as a confounder in this context. Ankylosis represents the most severe manifestation of HO. Some participants with (HO-associated) ankylosis had to be excluded from the study because essential neurological assessments were thus not feasible, which may have led to an underrating of the negative clinical impact of HO. Therefore, it is essential to use the current revision of the ISNCSCI in future studies, which indeed considers non-SCI conditions, thus preventing the loss of study participants due to missing data (Rupp et al., 2022). Nevertheless, HO as an already known relevant serious complication in daily clinical practice may additionally become a critical adversary in translating gains in neurological recovery—promoted through innovative regenerative therapies—into added functional improvement.

We found inferior functional outcomes for all subscales of the SCIM related to mobility, self-care, respiration, and sphincter management. A structural change in major joints such as the hip joints with a limited range of motion can obviously affect mobility, be it walking function or wheelchair mobility. However, impairment in functions related to self-care and sphincter management is not as obvious in this context. A closer look at respective SCIM items reveals that the performance in these categories also depends on the degree of assistance needed to manage these aspects of daily living successfully. Thus, impaired range of hip joint motion can indeed negatively impact respective SCIM subscales.

Both cohorts, which—based on the matching procedure—started with comparable UEMS and LEMS, did not show any difference in these parameters, even at the late stage. However, HO could theoretically have affected strength training, thus contributing to a less than optimal motor score. Alternatively, expanding bone formation could have compromised peripheral nerves (e.g., sciatic nerve) in the vicinity of the bone formation (Salga et al., 2015; Law-Ye et al., 2016; Onat et al., 2017), which was apparently not the case in the present study considering the similar outcomes regarding motor strength. Interestingly, the pin-prick assessment, reflecting pain sensation mediated by the spinothalamic tract, yielded lower scores in the HO group compared to the control group at both the early and the late stage. Of note, pin-prick scores were not part of the matching process. Reduced pain sensation could represent a risk factor for HO since individuals with SCI with severe paresis do not properly sense repetitive micro-traumatic impacts while practicing activities of daily living such as turning in bed, wheelchair transfer, or locomotor training (van Kuijk et al., 2002). This finding would need to be confirmed in a larger prospective study.

In this monocentric analysis, roughly 5% of the SCI individuals included in EMSCI at the SCI center Heidelberg between July 2002 and January 2020 had HO as documented diagnosis. The found incidence is lower than in previous publications (around 20%; Goldman, 1980). This discrepancy is probably due to a selection bias based on the retrospective character of the HO evaluation, the availability of complete datasets, and the fact that previous studies were characterized by a rather unselective inclusion of individuals with acute SCI (Lal et al., 1989; Krauss et al., 2015; Rawat et al., 2019). The rather high median age of 56 years in the HO cohort compared with previous reports (Wittenberg et al., 1992) is likely attributable to the same bias since eligible individuals with HO who had to be excluded were younger. Concerning other debated risk factors, the present individuals with HO represent a rather typical cohort: most of the individuals with HO in the study had marked injury severities (69% AIS A and B), a cervical level of injury (62%), with HO most frequently having occurred in the vicinity of the hip joints (85%) quite early after injury (median 56 days). Existing literature indeed reports SCI-related HO at the hips in about three-quarters to more than 90% of cases (Garland, 1988; Ohlmeier et al., 2017). Triggers for HO are most likely multifactorial, including genetic predisposition to insufficient mobilization early after the injury as well as microinjury to muscles and tendons in paretic limbs during active rehabilitative interventions (van Kuijk et al., 2002; Mitchell et al., 2010). Specific pathophysiological processes beyond inflammatory changes in the beginning and endochondral ossification as the end state are subject to ongoing research (Brady et al., 2018).

Considering the early development of HO after SCI and the serious functional consequences to be expected within the first year after injury, an early and stringent diagnostic and therapeutic regimen is highly desirable. However, in clinical practice, the management of HO is frequently rather heterogeneous due to a sparse evidence base resulting in a lack of effective clinical practice guidelines. None of the diagnostic measures such as basic laboratory diagnostic (alkaline phosphatase, C-reactive protein) or radiological workups (X-ray, CT, MRI, ultrasound, scintigraphy) alone or in combination allow diagnosing HO early on with a high level of confidence (May et al., 2000; Shehab et al., 2002; Estrores et al., 2004; Wick et al., 2005; Rosteius et al., 2017). While prophylactic administration of NSAR is already broadly established in clinical practice (Aubut et al., 2011), the potential to effectively prevent HO formation is limited. Radiation therapy is likely much more effective if applied in the early stage but requires a rather high degree of certainty in respect to the diagnosis considering potentially harmful side effects (Krauss et al., 2015; Museler et al., 2017; Yang et al., 2017). In the presented cohort radiation therapy was applied in more than three-quarters of cases. The detrimental impact of HO on functional outcome in the study cohort was most likely not prevented because radiation therapy was not administered early enough. HO resection surgery was performed in three individuals with severe manifestations of HO (Brooker stages of III or higher; Brooker et al., 1973)—towards the end of or beyond the 1-year-post-injury observation period. The impact of surgical HO resection to reverse or even worsen functional deficits cannot be determined, since a standardized functional assessment (SCIM) was not performed subsequently (Garland and Orwin, 1989; Meiners et al., 1997; Melamed et al., 2002; Genet et al., 2011). The effects of prolonged administration of rehabilitative interventions aiming to mitigate HO-induced functional deficits are unknown (Derakhshanrad et al., 2015). Results from the present study can inform the planning of future prospective studies probing early diagnosis and treatment regimens to effectively block function impairing HO formation by providing hints regarding clinically meaningful study endpoints and effect sizes.

### Limitations of the Study

EMSCI captures functional outcomes only up to 1 year after injury. Of course, differences in functional outcomes may have disappeared subsequently. Either more effective compensatory strategies or surgical resection of respective bone formations may have contributed to mitigating HO induced deficits.

The rather small sample size and the partly retrospective nature of this study (chart review to obtain information in respect to the diagnosis and treatment of HO) challenge the generalizability of this study. A recently implemented additional registry now provides a solid basis to prospectively record SCI-related secondary disease conditions such as HO (Rupp et al., 2021b). This eventually allows obtaining larger sample sizes combined with high-quality data to critically reflect the findings reported here and will help better understand the causes of deviating neurological and functional recovery profiles.

## Conclusion

Neurogenic heterotopic ossification represents a complication, which can add substantial secondary disability to the already grave neurological and functional deficits caused by SCI. HO-associated functional impairment as shown in the present study emphasizes the need for effective diagnostic and therapeutic measures, to tackle this condition as early as possible. Precious functional gains achieved through comprehensive SCI care and potentially augmented by effective restorative therapies, once they are available, are at stake.

## Data Availability Statement

The raw data supporting the conclusions of this article will be made available by the authors upon reasonable request, without undue reservation.

## Ethics Statement

The studies involving human participants were reviewed and approved by Ethics Committee, Medical Faculty of Heidelberg University, Alte Glockengießerei 11/1, 69115 Heidelberg, Germany. Phone: +49 62 21 56 2 64 6-0 Fax: +49 62 21 56 2 64 8-0 ethikkommission-I@med.uni-heidelberg.de. The patients/participants provided their written informed consent to participate in this study. Written informed consent was obtained from the individual(s) for the publication of any potentially identifiable images or data included in this article.

## Author Contributions

RR was significantly involved in the development and establishment of EMSCI. CS and SF were responsible for the study conception and design. LH, LR, RR, and SF the entire EMSCI study group were involved in the data acquisition. CS, LR, and SF contributed to data analysis and data interpretation. SF and NW drafted the manuscript. CS and LH drafted the Figures. CS, NW, and RR revised the final draft of the manuscript. All authors contributed to the article and approved the submitted version.

## Conflict of Interest

The authors declare that the research was conducted in the absence of any commercial or financial relationships that could be construed as a potential conflict of interest.

## Publisher’s Note

All claims expressed in this article are solely those of the authors and do not necessarily represent those of their affiliated organizations, or those of the publisher, the editors and the reviewers. Any product that may be evaluated in this article, or claim that may be made by its manufacturer, is not guaranteed or endorsed by the publisher.

## Abbreviations

AIS: ASIA Impairment Scale
AP: alkaline phosphatase
ASIA: American Spinal Injury Association
CRP: C-reactive protein
EMSCI: European Multicenter Study about Spinal Cord Injury
HO: heterotopic ossification
ISNCSCI: International Standards for Neurological Classification of Spinal Cord Injury
LEMS: lower extremity motor score
SCI: Spinal Cord Injury
SCIM: Spinal Cord Independence Measure
SL: sensory level
TLT: total light touch score
TPP: total pin-prick score
UEMS: upper extremity motor score.
