# Supplementary material for: Impact of Heterotopic Ossification on Functional Recovery in Acute Spinal Cord Injury
Source: Front Cell Neurosci. 2022 Feb 9;16:842090. doi: 10.3389/fncel.2022.842090 (PMC8864137; doi:10.3389/fncel.2022.842090)
Supplement: Supplementary file 1 [file Table_1.pdf]

Supplementary Table 1 Detailed comparison of sensory (sum) scores between individuals with heterotopic ossification and matched controls.

**Pair**	**Early stage of SCI**				**Late stage of SCI**			
	**TLT**		**TPP**		**TLT**		**TPP**	
	**HO**	**CON**	**HO**	**CON**	**HO**	**CON**	**HO**	**CON**
01	56	56	56	52	50	44	50	46
02	79	63	56	76	79	86	48	91
03	16	18	16	18	55	17	47	24
04	55	40	44	42	69	39	55	42
05	72	17	24	44	68	62	27	62
06	NA	42	NA	44	50	48	32	48
07	88	29	48	68	NA	46	NA	77
08	41	17	15	31	51	21	14	32
09	16	14	NA	14	18	12	9	38
10	16	95	NA	35	51	93	18	53
11	47	45	44	46	46	46	45	48
12	105	103	70	110	69	109	48	112
13	44	42	38	39	55	42	43	39
**Median**	**51**	**42**	**44**	**44**	**53**	**46**	**44**	**48**
(IQR)	(34.75-73.75)	(18-56)	(27,5-54)	(35-52)	(50-68,25)	(39-62)	(24,75-48)	(39-62)
**Mean**	**52.9**	**44.7**	**41.1**	**47.6**	**55.1**	**51.2**	**36.3**	**54.8**
(SD)	(±27.9)	(±27.6)	(±17.2)	(±24.4)	(±14.9)	(±28.3)	(±15.1)	(±24.0)
**p-value**	**0.87**		**0.004**		**0.58**		**0.02**	

Abbreviations: CON, controls; HO, heterotopic ossification; IQR, interquartile range; NA, not available; SCI, spinal cord injury; SD, standard deviation; TLT, total light touch; TPP, total pin-prick
